# A survey of mental health literacy in Japanese high school teachers

**DOI:** 10.1186/s12888-021-03481-y

**Published:** 2021-09-30

**Authors:** Satoshi Yamaguchi, Jerome Clifford Foo, Yuko Kitagawa, Fumiharu Togo, Tsukasa Sasaki

**Affiliations:** 1 Department of Physical and Health Education, Graduate School of Education, The University of Tokyo, 7-3-1 Hongo, Bunkyo-ku, Tokyo, 113-0033 Japan; 2 Central Institute of Mental Health, Medical Faculty Mannheim, University of Heidelberg, J5, 68159 Mannheim, Germany; 3 Center for Research on Counseling and Support Services, The University of Tokyo, 7-3-1 Hongo, Bunkyo-ku, Tokyo, 113-0033 Japan

**Keywords:** Adolescent, Child, Health education, Mental health literacy, School teacher

## Abstract

**Background:**

School teachers are well-positioned to recognize mental health problems in their students and to help them seek appropriate help. Therefore, teachers need to have high levels of mental health literacy (MHL). In East Asia, however, few studies have examined MHL levels in teachers. In this study, MHL levels were investigated in Japanese teachers.

**Methods:**

Teachers (*n* = 665) from 27 Japanese high schools answered a self-administered questionnaire which assessed (a) knowledge about mental health/illnesses, (b) correct recognition of specific illnesses (depression, schizophrenia and panic disorder), (c) confidence in helping students with depressive symptoms, and (d) confidence in teaching mental health knowledge to students.

**Results:**

The average proportion of correct answers to the knowledge questions (*n* = 20) was 58.1%. The proportion of those who correctly answered about the presence of a sharp increase of mental illnesses in adolescence was 51.7%. Few teachers correctly answered about the life-time prevalences of major mental illness in general (21.9%), depression (37.8%) and schizophrenia (19.8%). Depression, schizophrenia and panic disorder in vignette were correctly recognized by 54.1, 35.3 and 78.0% of teachers, respectively. Correct recognition was significantly lower in male than in female teachers. Only a small proportion of teachers had confidence in helping depressed students (19.9%) and in teaching mental health knowledge to students (11.1%).

**Conclusions:**

MHL in Japanese high school teachers appears to be low. Education programs should be developed and implemented to improve teacher MHL with the aim of helping them to support students suffering from mental health problems.

**Supplementary Information:**

The online version contains supplementary material available at 10.1186/s12888-021-03481-y.

## Background

The prevalence of mental illnesses increases sharply during adolescence [1, 2], but adolescents may have difficulty in recognizing their own mental health problems [3]; even when they can, they may be reluctant to seek help [4]. Considering that adolescents spend a major part of their time in schools, school teachers are uniquely positioned to recognize mental health problems in students and should be encouraged to support student help-seeking behavior [5]. To recognize these problems and provide support, teachers need to have sufficient knowledge of mental illnesses [6, 7]. Supporting behaviors of teachers are thought to be more frequent [6, 7] and more effective [8], when teachers have positive beliefs towards mental health problems and confidence in helping adolescents with the problems.

Knowledge and beliefs which aid in the recognition, management or prevention of mental health problems are defined as mental health literacy (MHL) [9]. MHL comprises several components, including the ability to recognize specific disorders, knowledge of risk factors and causes, and having attitudes that promote recognition and appropriate help-seeking [3, 9]. Thus far, a number of studies have observed that teacher-led MHL programs had positive impacts on MHL in adolescents [10–17], and schools are thought to be the best setting for sustainably delivering MHL programs to all students [18]. However, before teachers can deliver MHL programs, they must have sufficient MHL themselves.

A number of studies have examined teacher MHL in several countries and areas including Europe [19–22], the US [23–25] and Africa [26–29]. According to these studies, the majority of teachers had limited knowledge about mental health problems [25, 26, 29] and were not confident in helping students who were suffering from those problems [24]. Also, correct recognition of specific mental illnesses in vignettes was insufficient, especially in terms of schizophrenia and depression. Recognition was very low in several areas or countries (for instance, 33% for schizophrenia in certain regions in Italy [20] and 16% for depression in Nigeria [28]).

Meanwhile, few studies have investigated teacher MHL in Asia. In East Asia, only one study has examined MHL in teachers, assessing knowledge about schizophrenia in Japanese and Taiwanese primary school teachers [30]. The study observed that most surveyed primary school teachers could not correctly recognize schizophrenia in a vignette. MHL about depression and other mental health problems has not yet been examined in East Asian teachers.

In East Asia, most countries/areas have nation-wide examinations at the end of high school that determine entry into university [31–34]. These examinations make educational environments highly competitive, and a heavy emphasis on academic achievement causes academic stress, a major stressor in adolescents [33–37]. In addition, the average sleep duration of adolescents in East Asia is much shorter compared to other areas [38]; the competitive educational environment may contribute to this [36, 37]. The combination of stress and lack of sleep might lead to higher vulnerability to mental health problems in East Asian adolescents, a situation which requires more MHL from teachers.

The present study aimed to examine MHL in East Asian high school teachers. Knowledge about mental health/illnesses, the ability to recognize specific mental illnesses, and stigma towards mental illnesses were assessed.

## Methods

### Procedure and participants

A self-administered questionnaire (described below) was used to assess MHL in Japanese public high school teachers. In 2018, the Board of Education of Niigata prefecture (population: 2 million) organized a seminar for principals of all prefectural public high schools (*n* = 75). At the workshop, the principal investigator (T.S.) was asked to give a lecture regarding adolescent mental health. After the lecture, the Board of Education informed the principals about the current study using the Board website. The principals of 27 prefectural high schools told the Board that they wanted their schools to participate in the study, and 53.3% (*n* = 665) of teachers in those high schools participated. The teachers were asked to answer the questionnaire. The aim and contents of the study were explained to the teachers in writing. The teachers who participated provided written consent. The study was approved by The University of Tokyo Human Research Ethics Committee (#18–48).

### Contents of the questionnaire

MHL in teachers was assessed using a self-administered questionnaire. The questionnaire was written in Japanese, and comprised the following four parts.

#### Demographic variables

Demographic information of teachers was assessed, including age, sex, academic degree, previous participation in mental health seminars, and experience of dealing with someone suffering from a mental illness (Table 1).

**Table 1:** Demographic data of high school teachers

Characteristic	Number (%)
**Total**	665
**Age**	
20’s	35 (5.3%)
30’s	83 (12.5%)
40’s	239 (35.9%)
50’s	282 (42.4%)
60’s	17 (2.6%)
No answer	9 (1.4%)
**Sex**	
Male	451 (67.8%)
Female	175 (26.3%)
No answer	39 (5.9%)
**Academic degree**	
Associate degree^a^	34 (5.1%)
Bachelor	437 (65.7%)
Masters (or higher)	176 (26.5%)
No answer	18 (2.7%)
**Previous participation in MH seminars**	
None	429 (64.5%)
Once or more times	225 (33.8%)
No answer	11 (1.7%)
**Experience of dealing with someone suffering from a mental illness**	
No	191 (28.7%)
Yes	419 (63.0%)
No answer	55 (8.3%)
**If yes, who?**	
Student	376 (89.7%)
Friend	105 (25.1%)
Family	65 (15.5%)
Other	98 (23.4%)

*MH* mental health

^a^An associate degree is an undergraduate degree in Japan awarded after a course of post-secondary study lasting 2 or 3 years

#### General knowledge about mental health/illnesses

The second part of the questionnaire comprised 20 questions regarding general knowledge about mental health/illnesses (Table 2). The second part was developed as follows. The questionnaire consists of questions on basic knowledge about the epidemiology, symptoms and care/treatment of mental health problems/illnesses. Specific questions were made about the following issues, following several previous studies [1, 2, 39, 40] and the DSM-5 [41]: lifetime-prevalence of any major mental illnesses, sharp increase of mental health problems in adolescence, major symptoms and adverse effects on life of major mental illnesses, functions and durations of psychiatric treatments, adverse effects of alcohol on mental health, prevention of suicide, and more. The questionnaire was drafted by one of the authors (TS) and edited and refined by a team of psychiatrists, psychologists (including JCF), teachers (including SY) and school nurses. The possible answers to these questions were: “True”, “False”, or “I don’t know”. Correct answers were scored 1 (otherwise scored 0) and the scores were added up. The internal consistency (Cronbach’s alpha) of the 20 questions was 0.77 in the present sample.

**Table 2:** High school teachers’ knowledge about mental health/illnesses

Items	Correct answer	Proportion of correct responses (%)
The incidence of most mental illnesses sharply increases in adolescence.	T	51.7
About one in every 20 people will experience a mental illness.	F	21.9
Staying up late and lack of sleep influence the development of and worsen mental illnesses.	T	83.8
The duration of treatment for depression and schizophrenia is about half a year on average.	F	70.3
People with mental illnesses may only have somatic symptoms, including headaches, abdominal pain, and nausea.	T	65.3
When depressed mood, decreased motivation and diminished interest continue over time, it may be major depression.	T	89.4
In depression, both lack of sleep or insomnia, and oversleeping are possible.	T	76.2
People with mental illnesses may have difficulty riding vehicles (i.e. taking public transportation), leading to difficulties in attending school.	T	87.2
Using stimulant and illicit drugs is the major cause of auditory hallucinations and delusions.	F	62.4
Auditory hallucinations and delusions of being persecuted can be treated through talking.	F	41.2
More than 10% of people will experience depression.	T	37.8
Approximately 1% of people will experience schizophrenia.	T	19.8
Asking about suicidal ideation should be avoided, because it can lead to suicide attempts.	F	55.6
Students should return to school only after treatment for their mental illness has been completed.	F	56.1
When you cannot sleep, drinking alcohol can help you sleep better.	F	82.9
Drinking alcohol worsens anxiety and depression.	T	46.5
People with bipolar disorder are mostly identified when they are depressed.	T	24.7
Due to a mental illness, people may be unable to talk to others due to worry/nervousness.	T	82.0
In high school students, 7 h of sleep is ideal to decrease the risk of depression.	F	16.5
When you view bright lights late at night, you will have difficulty falling asleep.	T	90.5
Average percentage of correct answers to the knowledge questions		58.1

*F* false, *T* true

#### Recognition of and attitudes towards mental illnesses

Teachers were asked to read 3 case vignettes describing 3 teenage students with symptoms of depression, schizophrenia and panic disorder, respectively (Additional file 1). The vignettes were written by the authors, according to the DSM-5 criteria of the respective disorders [41].

##### Recognition of specific mental illnesses

Having read each vignette, teachers were asked “What is the name of the illness each student was suffering from?” The answer was selected from 6 choices: “no illness”, “depression”, schizophrenia”, “panic disorder”, “social phobia”, and “I don’t know”.

##### Stigma towards depressive symptoms

Three items regarding beliefs about the causes of symptoms which Student A in the depression vignette was suffering were adapted from the “weak-not-sick” subscale of the Depression Stigma Scale [42] (see Table 3) (One subscale item, “It is best to avoid people with a problem like Student A’s so that you do not develop this problem.” was excluded; the item was deemed as not appropriate for school teachers). Teachers were asked to what extent they agreed with the items. There were 5 answer choices: “Strongly agree”, “Agree”, “Neither agree nor disagree”, “Disagree”, “Strongly disagree”.

**Table 3:** Beliefs of high school teachers about causes of depressive symptoms in the vignette

Items	Proportions of responses (%)		
	Agree or strongly agree	Neither agree nor disagree	Disagree or strongly disagree
Student A could snap out of it if he/she wanted.	17.1	12.3	70.6
Student A’s problem is a sign of personal weakness.	6.1	14.8	79.1
Student A’s problem is not a real medical illness.	10.8	15.8	73.5

Note: Proportion of teachers who agree or strongly agree with any of the 3 items was 24.5%

##### Confidence in helping students with depressive symptoms

Teachers were asked, “How confident do you feel that you can appropriately help a student who is in a similar situation to Student A?” (student in the depression vignette). Possible answers to the question were “Fully confident”, “Confident”, “Not very confident”, “Not confident at all”, or “I don’t know”. The teacher was considered to have the confidence in helping the student, when the answer was “Fully confident” or “Confident”.

#### Confidence in teaching mental health knowledge to students

In the final section, teachers were asked “How confident do you feel if you are asked to teach a class about mental illnesses to students?” Possible answers to the question were “Fully confident”, “Confident”, “Not very confident”, “Not confident at all”, or “I don’t know”. The teacher was considered to have the confidence in teaching the knowledge, when the answer was “Fully confident” or “confident”.

### Statistical analysis

Demographic statistics were compiled and responses to the questionnaires were evaluated. Multiple regression analysis was conducted to examine whether demographic variables have effects on the knowledge score, while logistic regression analysis was conducted to examine whether they have effects on the correct recognition of specific mental illnesses. Listwise deletion was used for missing data on all variables. The level of significance was set at alpha = 0.05. The analyses were performed using R version 3.5.1.

## Results

### Demographic variables and general knowledge about mental health/illnesses

Table 1 shows demographic data of the teachers. Table 2 shows the proportion of teachers’ correct responses to the knowledge questions about mental health/illnesses. The average proportion of correct answers to the knowledge questions was 58.1% (standard deviation = 18.6%). Regarding specific questions, many items had low proportions (half or less than half) of correct answers, as follows: sharp increase of the incidence of most mental illnesses in adolescence (51.7%); life-time prevalence of any mental illnesses (21.9%), depression (37.8%), and schizophrenia (19.8%); adequate sleep duration to prevent depression in adolescents (16.5%); basic knowledge about the role of medication in schizophrenia (41.2%), adverse effect of alcohol on anxiety and depression (46.5%); and that people with bipolar disorder are mostly identified during depressive phases (24.7%).

### Recognition of specific mental illnesses and stigma towards depressive symptoms

Regarding the recognition of specific mental illnesses, 54.1, 35.3 and 78.0% of teachers correctly recognized depression, schizophrenia and panic disorder in the respective vignettes (not shown in Tables). Table 3 shows the beliefs of teachers about causes of depressive symptoms in the vignette. Approximately a quarter of teachers (24.5%) believed that depressive symptoms in the vignette were caused by personal weakness rather than a real medical illness.

### Confidence in helping students with depressive symptoms and in teaching mental health knowledge to students (not shown in tables)

Eighty percent of teachers (80.1%) answered that they did not have the confidence to help students suffering from depressive symptoms appropriately. Also, 88.9% of teachers answered that they did not have the confidence to teach mental health knowledge to students.

### Effects of demographic variables on knowledge about mental health/illnesses and correct recognition of mental illnesses

Table 4 shows the results of multiple regression analysis and logistic regression analyses. The proportion of correct recognition of schizophrenia was significantly lower among teachers in their 40s and 50–60s than 20–30s. Female teachers correctly recognized depression, schizophrenia, and panic disorder in vignette, significantly more than male teachers, while gender of teachers did not have a significant effect on the knowledge score. Previous participation in seminars addressing mental health in adolescents had a significant positive effect on the knowledge score, but not on the correct recognition of depression, schizophrenia, and panic disorder. Academic degree and experiences of dealing with someone suffering from a mental illness had no significant effect on correct recognition of all the disorders in vignette, or on the knowledge score.

**Table 4:** Differences of knowledge score and correct recognition of specific mental illnesses among high school teachers by their demographic data

Variable	Average percentage of correct answers to the knowledge questions (%)	Correct recognition (%)		
		Depression	Schizophrenia	Panic Disorder
**Age**				
20s & 30s (reference)	58.1	58.1	41.9	80.3
40s	57.3	55.5	37.0*	79.4
50s & 60s	58.7	51.0	31.8*	75.7
**Sex**				
Male (reference)	56.8	50.4	30.8	74.1
Female	60.1	61.5*	45.4**	87.9**
**Academic degree**				
Associate degree (reference)	61.1	50.0	32.4	73.5
Bachelor	57.7	52.8	35.3	78.6
Master	59.7	59.2	35.6	78.2
**Previous participation in MH seminars**				
No (reference)	55.7	51.9	31.7	76.3
Yes	62.8**	58.7	41.7	80.3
**Experience of dealing with someone suffering from a mental illness**				
None (reference)	54.5	50.3	25.9	74.1
1 or more time	59.8	56.8	38.8	80.3

*MH* mental health

Note: *P* values were derived from multiple regression and logistic regression analyses

*p < 0.05; **p < 0.01

## Discussion

To our knowledge, this is the first study to investigate MHL in high school teachers in East Asia (Japan). The teachers had limited knowledge about mental health and illnesses. The majority of teachers did not know about the high prevalence of mental illnesses, sharp increase of mental illnesses in adolescence, and optimal sleep duration for prevention of depression. Also, the majority of teachers did not correctly recognize depression and schizophrenia in the vignettes. In addition, few teachers felt confident in helping students with depressive symptoms, or in teaching mental health knowledge to students. This lack of knowledge, low recognition, and low confidence may lead to difficulty/inability in supporting students who have mental health problems.

Regarding the sharp increase of mental illnesses in adolescence, the majority of teachers seemed unaware that their students are at the age of the highest risk for mental illnesses. The onset of mental illnesses sharply increases in adolescence; 50% of all lifetime mental illnesses manifest by the age of 14 [1, 2]. This knowledge may be highly crucial for teachers in East Asia, considering that their students may be at high risk for mental health problems due to heavy stress arising from academic pressure [33–37]. It may be that a lack of teacher MHL can lead to delayed detection of mental illnesses and failure to administer a timely and appropriate intervention.

Most of the teachers agreed with the item stating that 7-h of sleep is appropriate to decrease the risk of depression in high school students. Teachers in East Asia may need to be educated about the optimal sleep duration to maintain good mental health for high school students, considering that the average sleep duration of high school students in East Asia is much shorter (approximately 6.5–7.0 h) [38] than suggested optimal sleep duration (8–10 h) [43]. Indeed, a Japanese study has observed a sleep duration of 8.5–9.5 h was associated with the lowest risk of depression/anxiety in male adolescents [44].

The majority of the participants did not correctly identify the names of illnesses in the vignette cases with depression and schizophrenia. The proportion of correct recognition was especially low for schizophrenia (35%), much lower than in several European countries (e.g. 60–68% in UK [20, 21], and 66–78% in Norway [22]). The low recognition of schizophrenia among the teachers should be improved, considering that the incidence of schizophrenia is very high in adolescence [39]. A potential reason for this low recognition may be linked to the fact that in Japan, schizophrenia was renamed in 2002 from “Seishin-Bunretsu-Byo” (English: mind-split-disease) to “Togo-Shitcho-Sho” (English: integration disorder) [45]. In addition, the proportion of correct identification was significantly lower among teachers in their 40s and 50–60s (37.0 and 31.8%, respectively) than in their younger counterparts; the renaming may have contributed to the lower proportion of correct recognition of schizophrenia in older teachers. This renaming of schizophrenia was also done in other countries/areas in East Asia (e.g., Korea, Taiwan, and Hong Kong) after the renaming in Japan [45]. Another potential reason for the low recognition may be linked to the state of the psychiatric care system in Japan; Japan had 2.61 psychiatric beds per 1000 persons in 2018, which was much higher than in other OECD countries (mean: 0.61 psychiatric beds per 1000 persons; range: 0.03–1.35) [46] and a figure which is largely unchanged over the last two decades (2.84 per 1000 persons in 1998) [46]. In addition, more than half (52.8%) of these beds are occupied by patients who have schizophrenia [46]. Lack of inclusion of patients into regular society may mean that Japanese people have fewer opportunities to communicate with schizophrenia patients than people in other countries, leading to lower recognition of schizophrenia by Japanese teachers.

The proportion of the correct recognition of schizophrenia was low and significantly lower in male teachers than female teachers. This gender difference was observed for all three illnesses, not only for schizophrenia. This may be in line with previous studies which observed the same trend in the general population [47–53]. In contrast to the gender difference, the level of education in teachers did not have a significant effect on knowledge score and recognition of specific mental illnesses. This may reflect that, in Japan, taking a mental health course is not required to obtain a teacher license in post-secondary school, including undergraduate and graduate school [54]. In addition, previous participation in mental health seminars did not have a significant effect on recognition of specific mental illnesses. A potential reason for no effect of mental health seminar may be linked to the fact that few MHL programs have been confirmed to be effective, and particularly in Japan, there have been no MHL program with confirmed effectiveness [55]. MHL programs for teachers need to be developed, with the confirmation of their effectiveness. Also, teachers’ experiences dealing with someone suffering from a mental illness did not have a significant effect on knowledge score and recognition of specific mental illnesses. Regarding the effect of previous experience, studies of teacher MHL have reported inconsistent results [19, 21]. Future studies need to investigate how teachers deal with someone suffering from a mental illness, as previous studies only investigate whether or not teachers had such experiences.

The current study has several limitations. First, participants were high school teachers from a single prefecture in Japan. Caution may be needed when generalizing results to other populations. As the present study is the first one from a single Japanese prefecture, further studies need to be carried out in different sites in Japan, as well as in East Asia. Second, it should be noted that the participation rate was not high (53.3%), which might reflect the busy schedules and heavy workloads of the teachers; a recent survey showed that Japanese teachers work the longest hours among all OECD countries [56]. Further research in more comprehensive samples is needed to confirm whether the present findings are representative of teachers in Japan. Third, the vignettes used in the current study were not validated; they were developed according to the DSM-5 criteria [41]. Fourth, we did not measure beliefs towards causes of symptoms of schizophrenia and panic disorder, nor the confidence in helping students with symptoms of these illnesses; future research will need to include these assessments.

## Conclusions

MHL in the Japanese high school teachers who participated in the present study was observed to be low. Teachers with low MHL may not notice mental health problems in their students, and even when they do, may have difficulty in helping them effectively. Knowledge about mental health/illnesses, and confidence in helping students may need to be improved in these teachers, so that they become able to notice the problems in students and support their help-seeking behaviours appropriately [8]. Improvements in MHL may also enable the teachers to confidently teach mental health knowledge to students. Presently, although a number of MHL programs targeting teachers have been developed, only a few have been confirmed to be effective [55]. Programs for teachers with confirmed effectiveness need to be developed. Such programs may also need to be incorporated into teacher education at the university level, because in most East Asian countries/areas in addition to Japan, the inclusion of MHL is not currently standard [54, 57–59].

## Supplementary Information

**Additional file 1: Additional Table 1.** Vignettes for depression, schizophrenia, and panic disorder.

## Data Availability

The data that support the findings of this study are available from the corresponding author upon reasonable request.

## Abbreviation

MHL: Mental health literacy
