# Supplementary material for: A survey of mental health literacy in Japanese high school teachers
Source: BMC Psychiatry. 2021 Sep 30;21:478. doi: 10.1186/s12888-021-03481-y (PMC8482625; doi:10.1186/s12888-021-03481-y)
Supplement: Supplementary file 1 — Additional file 1: Additional Table 1. Vignettes for depression, schizophrenia, and panic disorder. [file 12888_2021_3481_MOESM1_ESM.docx]

Additional Table 1. Vignettes for depression, schizophrenia, and panic disorder

| **Depression vignette**  Student A goes to the health care room in the school, reporting having a headache and stomachache, and feeling tired. Student A has trouble sleeping, doesn’t feel like eating, doesn’t have fun watching his/her favorite TV program, and can’t keep his/her mind on his/her studies. Student A is often late for school these days. |
| --- |
| **Schizophrenia vignette**  Student B appears to have trouble in concentrating in class, compared to before. Student B covers his/her ears during break time. When asked, Student B says, “I feel that someone is always spying on me. People in class are always saying bad things about me/talking about me behind my back. When strangers pass by, I feel like they are also saying bad things about me. I feel nervous and concerned about noises and voices in the surroundings.” |
| **Panic disorder vignette**  In the bus on the way to school, Student C sometimes suddenly feels his/her heart pounding and has difficulty in breathing. When this happens, cold sweats and trembling do not stop, and Student C feels scared that he/she will suddenly die. Due to fear of this happening again, Student C became unable to take the bus. |
